# Evolutionary Reconstruction and Population Genetics Analysis of Aurora Kinases

**DOI:** 10.1371/journal.pone.0075763

**Published:** 2013-09-24

**Authors:** Balu Kamaraj, Ambuj Kumar, Rituraj Purohit

**Affiliations:** Bioinformatics Division, School of Bio Sciences and Technology, Vellore Institute of Technology University, Vellore, Tamil Nadu, India; The Scripps Research Institute, United States of America

## Abstract

**Background:**

Aurora kinases belong to the highly conserved kinase family and play a vital role in cell cycle regulation. The structure and function of these kinases are inter-related and sometimes they also act as substitutes in case of knockdown of other aurora kinases.

**Method:**

In this work we carried out the evolutionary reconstruction and population genetic studies of aurora kinase proteins. Substitution saturation test, CAI (Codon adaptation index), gene expression and RSCU (Relative synonymous codon usage) values were computed for all the three aurora kinases. Linear regression method was used to check the dependency of gene expression on their CAI values.

**Results:**

The results suggested that aurora-B and aurora-C has shown convergence in their evolutionary pathway. Moreover, the aurora-A I57V mutation showed high penetrance in human population and exist at very high frequency (84.4%) when compared to the native residue (15.6%). The mutation showed notable range of functional gain and seemed to be promising for the evolution of aurora-A function. Mutant allele might also become a challenging prospect for understanding the pattern of evolution followed by cell cycle kinases.

**Conclusion:**

The overall result suggested that the aurora-A is currently under the evolutionary transition and to determine the functional significance of the mutation further investigation are required.

## Introduction

Aurora kinases belong to the highly conserved protein kinase family and play a vital role in regulating cell cycle progression mechanism. They help the dividing cell to dispense its genetic materials to its daughter cells. More specifically, Aurora kinases play a crucial role in cellular division by controlling chromatid segregation [1]. The activity of Aurora kinases are strictly regulated by a group of events like as phosphorylation and degradation. Special interest in Aurora kinases has arisen since people discovered their defects in association with severe mitotic abnormalities and cancers. Deregulation of Aurora kinase activity can result in mitotic abnormality and genetic instability, leading to defects in centrosome function, spindle assembly, chromosome alignment, and cytokinesis [2]. The human aurora family of serine-threonine kinases comprises three members, which act in concert with many other proteins to control chromosome assembly and segregation during mitosis [3]. Aurora A expression in tumours is often associated with gene amplification, genetic instability, poor histologic differentiation, and poor prognosis [1]. Aurora B is frequently expressed at high levels in a variety of tumours, often coincidently with aurora A, and expression level has also been associated with increased genetic instability and clinical outcome [1]. Further, aurora kinase gene polymorphisms are associated with increased risk or early onset of cancer. The expression of aurora C in cancer is less studied. In recent years, several small aurora kinase inhibitor molecules have been developed that exhibit preclinical activity against a wide range of solid tumours [3].

Aurora kinases comprise mainly two domains: a regulatory domain in the NH2 terminus and a catalytic domain in the COOH terminus. The regulatory domain is largely diverse, whereas the catalytic domain with a short segment of diverse COOH terminus shares >70% homology among Aurora-A, Aurora-B, and Aurora-C. There is a D-Box in the COOH terminus and an A-Box in the NH2 terminus of Aurora kinases, which are responsible for degradation [2,4-7]. These features have been confirmed by experiments in both Aurora-A and Aurora-B; yet, the structure of Aurora-C is deduced by sequence alignment and seems to lack the A-Box. Aurora-A and Aurora-B are distinct in subcellular distribution during mitosis [2]. Despite great similarities in sequences and structures, Aurora kinases are completely different in their subcellular distribution. Aurora-A localizes to the pericentriolar material from the end of S phase to the beginning of the next G1 and spreads to the pole proximal ends of spindle microtubules during mitosis. In contrast, Aurora-B remains in the nucleus and moves to centromeres from prometaphase to metaphase [2]. After anaphase begins, Aurora-B relocates gradually to the midzone and persists at the midbody until cytokinesis is completed. Moreover, Aurora-B undergoes different localizations along with at least three other partners: INCENP, Survivin, and Borealin [2]. They form a tight complex within the cell during mitosis and are named “chromosomal passengers” as they move precisely from site to site at specific times. By G2 phase, Aurora-A localizes to pericentriolar material and persists throughout cell cycle. Additionally, it spreads to the minus ends of the mitotic spindle microtubules and midzone microtubules during mitosis [2]. Diversely, Aurora-B and its “passenger” partners remain a part of centromeres from prometaphase to metaphase. After chromatids begin to separate, they relocate to midzone and persist at the midbody until cytokinesis is completed. Moreover, Aurora-A and Aurora-B exhibit divergent functions in mitotic control, corresponding to different subcellular distribution of the two kinases. Aurora-A is mainly involved in centrosome function, mitotic entry, and spindle assembly, whereas Aurora-B participates in chromatin modification, microtubule-kinetochore attachment, spindle checkpoint, and cytokinesis. Along with these two kinases, different partners and substrates take part in these processes [2]. But in contrast to the wide range of distinct variation in their functional regulation, when N158 is mutated to G, the characteristic amino acid at that position in Aurora A, Aurora B became 350-fold more active than wild-type enzyme, similar to the activity level of wild type Aurora A [8]. Also it is remarkable that the large difference in intrinsic activity of the two kinases in the absence of regulatory subunits can be account for a single amino acid difference [8]. It seems as if by compromising at some specific amino acid position cause greater convergence in the activity regulation of Aurora-A and Aurora-B which explains the interlinking between their evolutionary pathways.

Although some vital experiments have handsomely explained the function of Aurora-C, the explicit insights have not been obtained. It has been suggested that Aurora-C is a Serine/threonine-protein kinase component of the chromosomal passenger complex (CPC), a complex that acts as a key regulator of mitosis. The CPC complex has essential functions at the centromere in ensuring correct chromosome alignment and segregation and is required for chromatin-induced microtubule stabilization and spindle assembly. It also plays a vital role in meiosis and more particularly in spermatogenesis. It has redundant cellular functions with Aurora B and can rescue an Aurora B knockdown. Like Aurora B, Aurora C phosphorylates histone H3 at 'Ser-10' and 'Ser-28'. It also phosphorylates TACC1, another protein involved in cell division, at 'Ser-228' [9-11]. It is likely to be understood that the Aurora-B and Aurora-C shares significant functional coordination and thus it is likely to be evolved in convergent manner. Also, by observing the traces of evolutionary linkage between the Aurora-A and Aurora-B proteins, it is evident that all three Aurora kinases might have shared some common evolutionary road map.

In this study we have investigated the evolutionary relationship between the three aurora kinases and compiled the crucial factors that might have governed their evolutionary pattern. Phylogenetic reconstructions were conducted to study their evolutionary divergence. Furthermore, the sequence based analyses were carried to investigate the functional regions evolving under different rates and also crucial factors governing their evolution. Moreover the population genetic studies were conducted to determine the substitution based evolution occurring in the aurora kinases in present human population. The overall results obtained from this study would aid in determining the key amino acid residues and the regions involved in functional regulation of aurora kinases.

## Materials and Methods

### Datasets

The coding sequence data for Aurora kinases were retrieved from the ENSEMBLE/GenBank gene databases [12]. We considered the protein coding mRNA sequence (CDS) by removing the partial and internal stop codon sequences. Partial and the incomplete sequences were omitted from the dataset.

### Phylogenetic Reconstruction

We constructed phylogenetic trees using distance maximum parsimony (MP), maximum likelihood quartet puzzling (QP) and Bayesian posterior probabilities (BP). The programs SEQBOOT and CONSENSE were used to estimate the confidence limits of branching points from 1000 bootstrap replications. ML tree topologies were constructed using the software PUZZLE 4.0 [13], employing 1000 puzzling steps, the JTT substitution matrix, estimation of rate heterogeneity using the gamma distribution model with eight rate categories, and the gamma-parameter estimation from the dataset. MP analysis was performed using PAUP4.0b5 software [14] where the number and lengths of minimal trees were estimated from 100 random sequence additions, while confidence limits of branch points were estimated by 1000 bootstrap replications. BP trees were constructed using the software MrBayes v3.0B4 [15,16]. Bayesian analysis used the mixed model of sequence evolution with random starting trees. Markov chains were run for 106 generations, burn-in values were set for 104 generations, and trees sampled every 100 generations. All trees were visualized using the program Dendroscope [17]. The multiple sequence alignment for the Aurora kinase proteins were obtained using CLUSTALW [18].

### Substitution saturation and relative synonymous codon usage analysis

Substitution saturation in all three aurora kinase protein coding region was determined by using DAMBE (data analysis in molecular biology and evolution) software V.5.2.9 [19,20]. The relative synonymous codon usage (RSCU) [21] method was applied to calculate the codon usage for the dataset. Relative synonymous codon usage (RSCU) is defined as the ratio of the observed frequency of codons to the expected frequency given that all the synonymous codons for the same amino acids are used equally. RSCU values have no relation to the amino acids usage and the abundance ratio of synonymous codons, which can directly reflect the bias of synonymous codon usage [22].

### CAI and Expression analysis

The codon adaptation index (CAI) was used to estimate the extent of bias towards codons that were known to be preferred in highly expressed genes [21]. It is now proved that CAI values mostly approach the theoretical values to reflect the expression level of a gene. Thus it has been widely utilized to measure the gene expression level [23,24]. A CAI value ranges from 0 to 1.0, and a higher value indicates a stronger codon usage bias. CAI provides an indication of gene expression level under the assumption that there is translational selection to optimize gene sequences according to their expression levels. Relationship between the evolution of codon usage pattern and the corresponding gene expression values were computed using DAMBE package. Linear regression analyses were conducted to examine the dependence of expression on the CAI values of aurora kinases. The dependencies were computed using the standard linear regression model given below.

α=βx+ε

Here α is the dependent variable (expression value) and the β is the independent variable (CAI). The x is the slope and ε is the intercept of the line on Y axis.

### Positive selection detection

PAML [25] and HyPhy [26] packages were implemented to compute the positively selected residues in Aurora kinases. The type of selection constraint for the identified sites can be tested based on the posterior probability values. The inferred codon sites with significant posterior probability (>50%) are likely to be under variable selection pressure as estimated by the Bayes empirical Bayes (BEB) and naive empirical Bayes (NEB) approach. Codeml models of PAML (phylogenetic analysis maximum likelihood) package were applied to predict the codon sites and the type of selective pressure in a protein-coding sequence, as different codon would be under different selective pressures [27]. The models compute the rate of nucleotide substitution per codon site using non-synonymous (amino acid changing, dN) to synonymous (no change in amino acid, dS) ratio (ω) which indicates the selective pressure that is inferred through maximum likelihood method. Furthermore, the Random Effect Likelihood (REL) program from HyPhy package was implemented to examine the positively selected residues in Aurora kinases. It is a reliable model for low divergence or fewer numbers (5 to 15) of sequences for conducting a selection test. The program automatically screens the submitted set of aurora kinase sequences to identify the best nucleotide substitution models. These models were computed using hierarchical testing procedure together with LRT and AIC. The REL codon site model was fitted to the aurora kinase subclass genes by incorporating the nucleotide substitution model with the generated NJ trees. For every codon site, REL computes two Bayes factor values; if the computed value for a site was >50, then the amino acid sites are said to be positively selected otherwise negatively selected. Finally both PAML and HyPhy packages were used altogether to compute the dN/dS ratios to predict the most likely position of substitution selection.

### Population genetics studies

SPSmart [28] was used to examine the penetrance level of Aurora kinases large scale genomic mutations in different human populations. The SPSmart engine encompasses a range of data sets: Hap-Map release, the Stanford University and University of Michigan CEPH-HGDP, i.e., Centre d’Etude du Polymorphisme Humain (CEPH) Human Genome Diversity Panel (HGDP) and the Perlegen SNP Data Set [28]. The mutations with penetrance frequency > 50% was considered as significant. Further, the mutations were subjected to SNAP (screening for non-acceptable polymorphisms) package [29] which is a neural network-based method for the prediction of the functional effects of non-synonymous SNPs and used to examine its linkage disequilibrium values. MutPred [30], SNPeffect 4.0 [31], SIFT [32], Polyphen 2.0 [33] and I-mutant2.0 [34] tools were implemented to study the molecular changes associated with the above compiled mutations. MutPred is a web based tool, used to predict the molecular cause of disease related amino acid substitution [30]. It utilizes several attributes related to protein structure, function, and evolution. Associated phenotype changes for the predicted mutation were examined using SNPeffect4.0 tool [31]. SIFT uses sequence homology-based approach to classify amino acid substitutions [32]. The prediction score < 0.05 is considered to be deleterious. PolyPhen 2.0 is based on combination of sequence and structure based attributes and uses naive Bayesian classifier for the identification of an amino acid substitution and the impact of mutation. The output levels of probably damaging and possibly damaging were classified as functionally significant (≤0.5) and the benign level being classified as tolerated (≥0.51) [33]. I-Mutant 2.0 is a support vector machine (SVM) based tool for the automatic prediction of protein stability changes upon single point mutations. I-Mutant 2.0 can be used both as a classifier for predicting the sign of the protein stability change upon mutation and as a regression estimator for predicting the related DDG values [34]. From the above tools, it was clearly showed the molecular changes upon mutations.

## Results and Discussion

**Figure 1:**
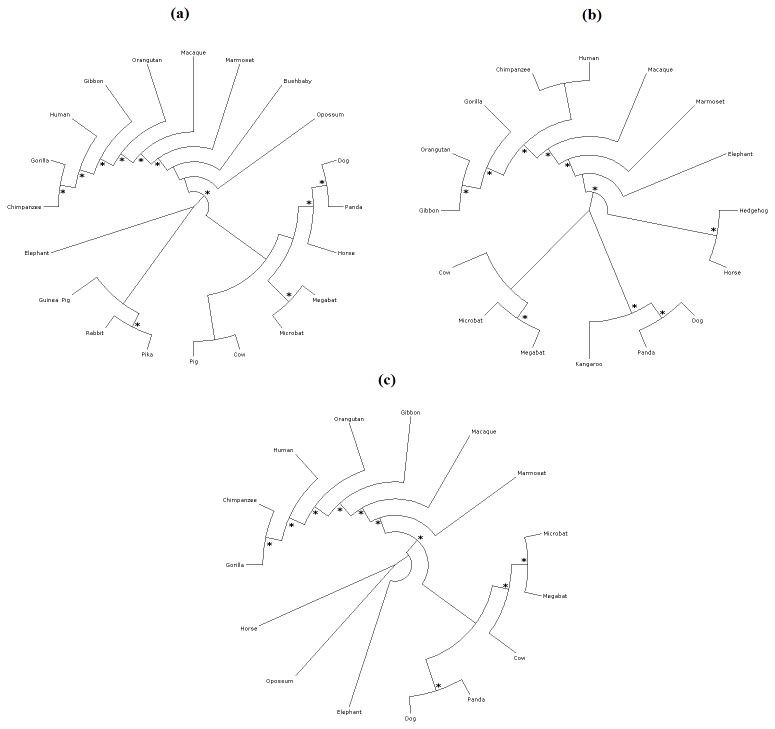
Reconstructed phylogenetic tree of Aurora kinase protein. Asterisks ("*") indicate those nodes supported 70% or greater by distance maximum parsimony (MP) and maximum likelihood quartet puzzling (QP) tree-building methods and 0.90 Bayesian posterior probability. (a) Aurora-A, (b) Aurora-B, (c) Aurora-C.

**Figure 2:**
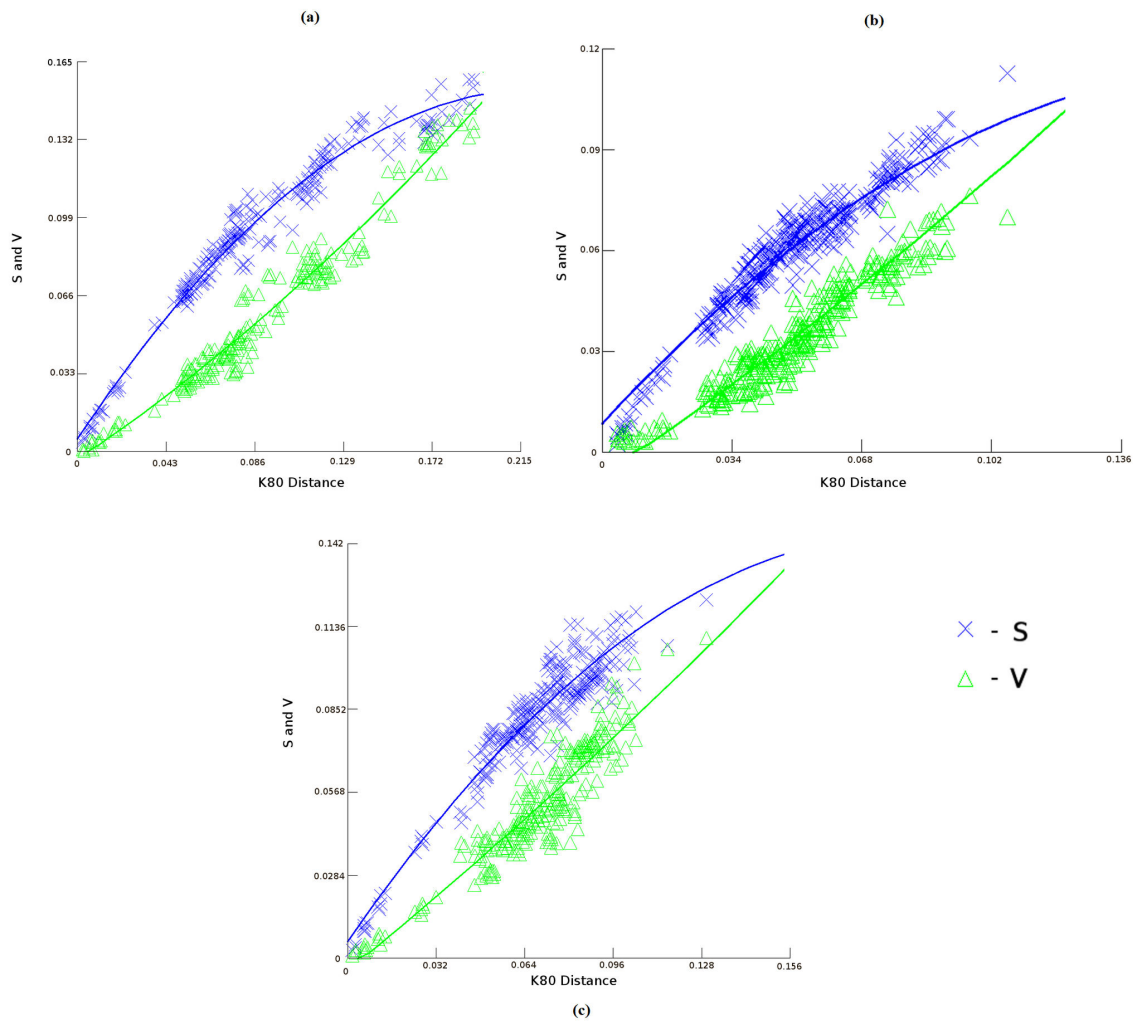
Transition (S) and transversion (V) values plotted against the K80 distance for (a) Aurora-A, (b) Aurora-B, (c) Aurora-C.

Nucleotide sequence for the coding region of Aurora kinases were collected from the ENSEMBLE database specifically for the primate species. Since some of the primate species Aurora kinases are not completely sequenced, we omitted them from our study. Moreover, the coding sequence of rat and mouse were not included due to the presence of additional functional motiff in their coding region which might cause errors while calculating the substitution saturation rates. Furthermore, few species such as Bushbaby, Pika, Rabbit and Guinea pig sequences were not included while constructing the evolutionary tree for Aurora kinase B and C due to the unavailability of nucleotide sequence for their complete coding region in these species. Multiple sequence alignments showed that the Aurora kinase family is highly conserved. Pairwise sequence comparisons had suggested that the mean proportion of similar amino acids is much higher among all the different families of Aurora-A, Aurora-B and Aurora-C of vertebrates than within the same family (Aurora-A or Aurora-B) between vertebrates and invertebrates species. This suggested a recent evolutionary radiation of Aurora families within vertebrates [35]. Moreover, the phylogenetic tree shown in Figure 1 suggested that the Aurora kinases had followed a similar evolutionary road map. We further conducted substitution saturation test, to investigate if the proteins had reached the level of substitution saturation threshold level. The transition transversion plots against the K80 distances clearly showed that the evolution driven substitution in aurora kinase proteins were well under the saturation threshold level (Figure 2). Moreover, a greater divergence between the transition-transversion rates were observed in case of Aurora-A, depicting that the Aurora-A is more likely to evolve when compared to Aurora-B and Aurora-C (Figure 2).

The evolution driven changes in the codon usage pattern significantly accounted for the distinct functional behaviour existing within the proteins. The results obtained in our RSCU analysis further suggested a supportive evidence of multiple codon usage patterns among the aurora kinases. It was interesting to see that the codons such as GCU, UGC, GAU, GGA, CUG, UUG, AUG, CCU, CCA, AGA, UCU, ACC and UGG were shown to exist at very high usage frequency (Table 1). It made sense to say that these codons might construct the signature patterns of Aurora kinase family. Moreover, there were several other codons which were showing higher usage frequency specific to the individual protein. Furthermore, it was interesting to see that the codon triplets used for coding serine amino acids did not show high usage frequency in Aurora-B and Aurora-C, whereas in Aurora-A, the frequency of serine coding triplets showed relatively higher usage pattern. This might account for its specific scaffolding behaviour, allowing their amino acid residues to be phosphorylated in a different manner.

**Table 1:** RCSU value of codons for AURKA, AURKB and AURKC genes.

Amino Acid	CODON		RCSU	
		AURKA	AURKB	AURKC
ALA	**GCU**	**1.202**	**1.380**	**1.121**
	GCC	0.875	1.023	1.067
	GCA	1.503	0.865	1.058
	GCG	0.421	0.732	0.754
CYS	UGU	0.906	0.678	0.750
	**UGC**	**1.094**	**1.322**	**1.250**
ASP	**GAU**	**1.294**	**1.629**	**1.112**
	GAC	0.706	0.371	0.888
GLU	GAA	1.322	1.403	0.871
	GAG	0.678	0.597	1.129
PHE	UUU	0.993	1.245	1.024
	UUC	1.007	0.755	0.976
GLY	GGU	0.968	0.650	0.577
	GGC	0.758	0.782	0.694
	**GGA**	**1.675**	**1.606**	**1.215**
	GGG	0.599	0.962	1.514
HIS	CAU	0.934	1.050	0.964
	CAC	1.066	0.950	1.065
ILE	AUU	1.482	0.846	1.065
	AUC	1.042	1.254	0.935
	AUA	0.476	0.900	0.619
LYS	AAA	1.298	1.164	0.671
	AAG	0.702	0.836	1.329
LEU	CUU	0.995	1.489	0.878
	CUC	0.885	0.370	0.780
	CUA	1.000	1.072	0.618
	**CUG**	**1.121**	**1.069**	**1.724**
	UUA	0.917	0.961	0.528
	**UUG**	**1.083**	**1.039**	**1.472**
MET	**AUG**	**1.000**	**1.000**	**1.381**
ASN	AAU	0.860	1.076	1.179
	AAC	1.140	0.924	0.821
PRO	**CCU**	**1.440**	**1.326**	**1.197**
	CCC	0.849	1.068	1.201
	**CCA**	**1.372**	**1.097**	**1.161**
	CCG	0.340	0.510	0.440
GLN	CAA	1.372	1.346	0.846
	CAG	0.931	0.654	1.154
ARG	CGU	0.814	0.766	0.709
	CGC	0.945	0.574	0.880
	CGG	0.989	1.313	1.547
	CGA	1.252	1.347	0.863
	**AGA**	**1.398**	**1.094**	**1.384**
	AGG	0.602	0.906	1.757
SER	**UCU**	**1.027**	**1.510**	**1.394**
	UCC	1.323	0.861	1.429
	UCA	1.313	0.970	0.806
	UCG	0.338	0.660	0.371
	AGU	1.195	1.121	0.736
	AGC	0.805	0.879	1.264
THR	ACU	1.296	0.936	0.675
	**ACC**	**1.046**	**1.406**	**1.293**
	ACA	1.215	0.872	1.434
	ACG	0.443	0.786	0.598
VAL	GUU	0.998	0.927	0.736
	GUC	1.449	1.201	0.643
	GUA	0.850	0.952	0.834
	GUG	0.702	0.920	1.787
TRP	**UGG**	**1.000**	**1.000**	**1.193**
TYR	UAU	1.132	0.957	0.945
	UAC	0.868	1.043	1.055

Codons highlighted in bold were having RCSU values greater than 1 in all three genes.

**Figure 3:**
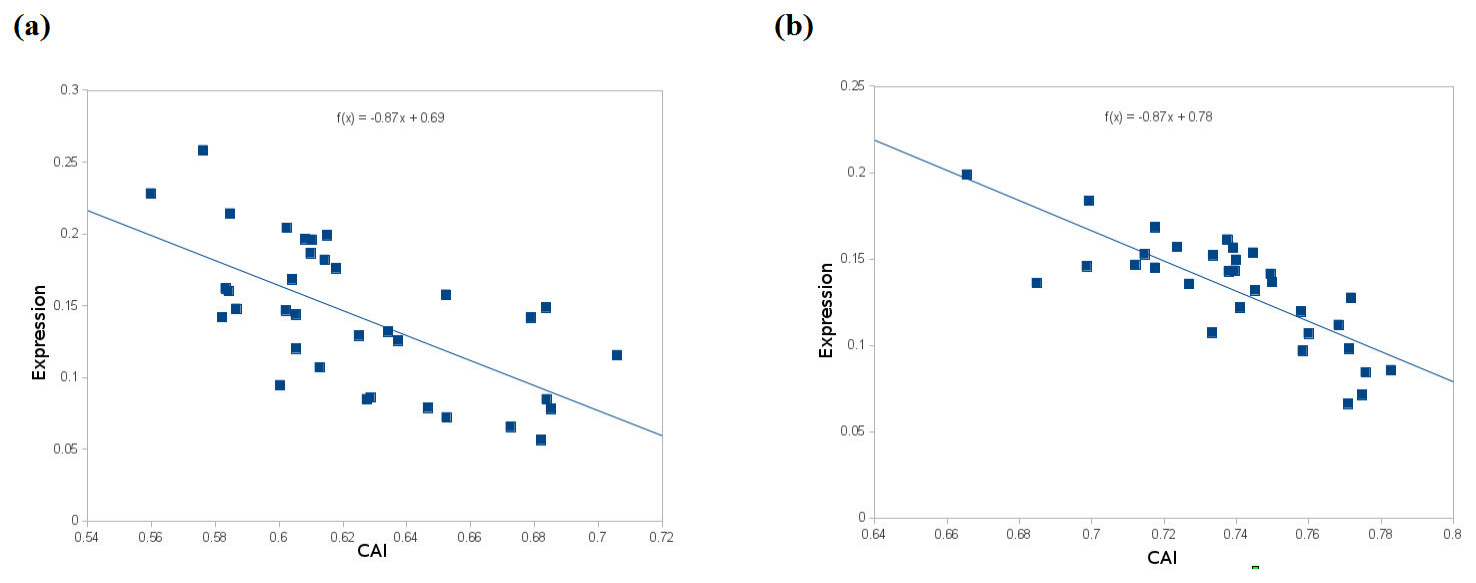
Gene expression values plotted against the codon adaptation indices. (a) Aurora-B, (b) Aurora-C.

Evolution can affect the phenotype either by modifying the sequences of proteins or by changing their pattern of expression. Although there were evidences of evolution showing co-regulatory behaviour on the gene expression level and the corresponding codon sequences. Thus, we further extended our study to examine if these variations in the codon usage pattern caused any changes in its expression patterns. CAI is a widely used index for characterizing gene expression in general and translation efficiency in particular. The CAI values for each of these genes were compiled and plotted against their expression values obtained from in-silico experiment (Figure 3). The expression values obtained in this study were calculated using the isoelectric gel profiling method included in DAMBE package, which has been trained on the empirical data’s of acid-resistant gastric pathogen *Helicobacter pylori*. The pI values from DAMBE using the same method had also been used to study the adaptive evolution of the matrix extracellular phosphoglycoprotein in mammals and the implication of its change on protein folding [36]. A notable pattern of gene expression changes and their corresponding CAI values were observed for Aurora-B and Aurora-C, whereas for Aurora-A there was no strict fashion of CAI dependent expression changes. The plot clearly depicted that the CAI had greater role in optimizing the expression values of Aurora-B and Aurora-C genes (Figure 3). With the rise in CAI values, the gene expression reduced consistently and extended up to ~ 0.06 in both the genes. Following dependencies were observed for Aurora-B.

Expressionvalue=−0.874×CAI+0.78

whereas for Aurora-C, the dependency was

Expressionvalue=−0.873×CAI+0.69

In aurora-A, no such uniform and strict regression patterns were observed, suggesting that the Aurora-B and Aurora-C showed a similar fashion of CAI based gene expression changes.

The extent to which selection affected genes and genomes is a key question in genetics and molecular evolution. Selection might modulate gene sequence evolution in different ways, for example, by constraining potential changes of amino acid sequences (purifying or negative selection) or by favouring new and adaptive genetic variants (positive selection) [37]. To quantify selection in the simplest case, the number of non-synonymous differences in a pair of protein-coding sequences could be estimated. However, substitution rates vary across the genome and between species that made direct comparisons solely based on non-synonymous substitutions difficult. To control variation in the underlying mutation rate, a standard way had to take the ratio of the number of non-synonymous differences per total number of possible non-synonymous changes (dN) to the number of synonymous differences per total number of synonymous changes (dS) [38]. This ratio (dN/dS) was then used as a measure of ‘‘functional divergence’’ that accounted for the underlying local or regional variation in the substitution rate for which dS is taken as a proxy. The ratio dN/dS were calculated using PAML and HyPhy packages. It was interesting to observe that all three aurora kinases mainly evolved under the influence of negative selection showing 58 negatively selected sites in Aurora-A, 64 in Aurora-B and 93 negatively selected sites in Aurora-C protein coding region. At any given moment of time, positive selection, which favoured currently uncommon derived alleles, affected only a small fraction of sites in the genome and, thus, was much rarer than negative selection, which favoured common ancestral alleles [39]. Since the higher number of neucleotide substitutions were very harmful for the conserved proteins, specially the aurora kinases, and the negative selection played an important role in maintaining the long-term stability of their structures and function by removing deleterious mutations. When the required optimizations were achieved, there was a danger of losing that improvement by a deleterious mutation. The negative selection made sure that deleterious mutations could not take over a population and that any improved structures, once fixed in a population, were maintained as long as they were needed [40]. The specificity of scaffolding in aurora kinases requires evolution driven optimization of their structure which further made the negative selection as the key criteria for its evolution. The substitution saturation results suggested that more or less all the three aurora kinase structures were tending towards their required optimization levels and hence any further substitution was most likely to cause major deleterious effect in their structure as well as function.

Positive selection had undoubtedly played a critical role in the evolution of *Homo sapiens* and promoted the emergence of new phenotypes. Comparative genetics/genomics studies in recent years have uncovered a growing list of genes that might have experienced positive selection during the evolution of human and/or primates. Moreover we investigated the positively selected residues that could have played significant role in evolution of aurora kinases function. PAML predicted a total of two residue positions including 31F and 66S in Aurora-A, 17A, 69T, 68L, 294A and 300A in Auora-B and 5R, 12K, 13A, 26T and 33P in Aurora-C. REL model predicted 23K, 31F and 66S in Aurora-A as positively selected, 16T, 17A, 69T, 294A and 300A in Aurora-B as positively selected, whereas 5R, 7V, 12K, 13A, 26T and 245S as positively selected in Aurora-C. Significance of these selections could be directly seen, where the Aurora A (F31I) polymorphism showed preferential amplification associated with increased aneuploidy in colon cancers and was a low-penetrance cancer susceptibility allele affecting multiple cancer types. Residues position 31F was shown to be essential for the binding of E2 ubiquitin-conjugating enzyme (UBE2N) protein, resulted in co-localization of UBE2N with Aurora-A at the centrosomes during mitosis. The 31I variant showed notable loss in the binding potential, ultimately leading to the degree of aneuploidy in human colon tumours [41]. This directly indicates the role of positive selection in optimizing the function of Aurora-A protein, which in turn aids in its essential interaction with UBE2N protein. Furthermore, the protein kinases exert control over their protein targets by covalent modification of a Ser, Thr, or Tyr residue with the γ-phosphate group cleaved from ATP. The selection of 66S in Aurora-A, 16T and 69T in Aurora-B and 26T and 245S in Aurora-C might correspond to an additional phosphorylation sites required for their recruitment and scaffolding activity. Moreover, the distribution of positively selected residues suggested that the majority of selections were concentrated in the N terminal flanking region. The N-terminal region determines selectivity during protein-protein interactions [42]. The N lobe was responsible for positioning the ATP phosphate group through a αC helix, whereas the activation loop within the C lobe was able to harbour substrates. Only when the two lobes arrive at a certain conformation could the Aurora kinases fulfil their kinase functions. Low conservation and high evolvability in this region might be a crucial factor in the evolution of complex network of its scaffolding activity. Although the clear picture of their functional role had not been established, which was likely be essential for maintaining the activity of the aurora kinases.

**Figure 4:**
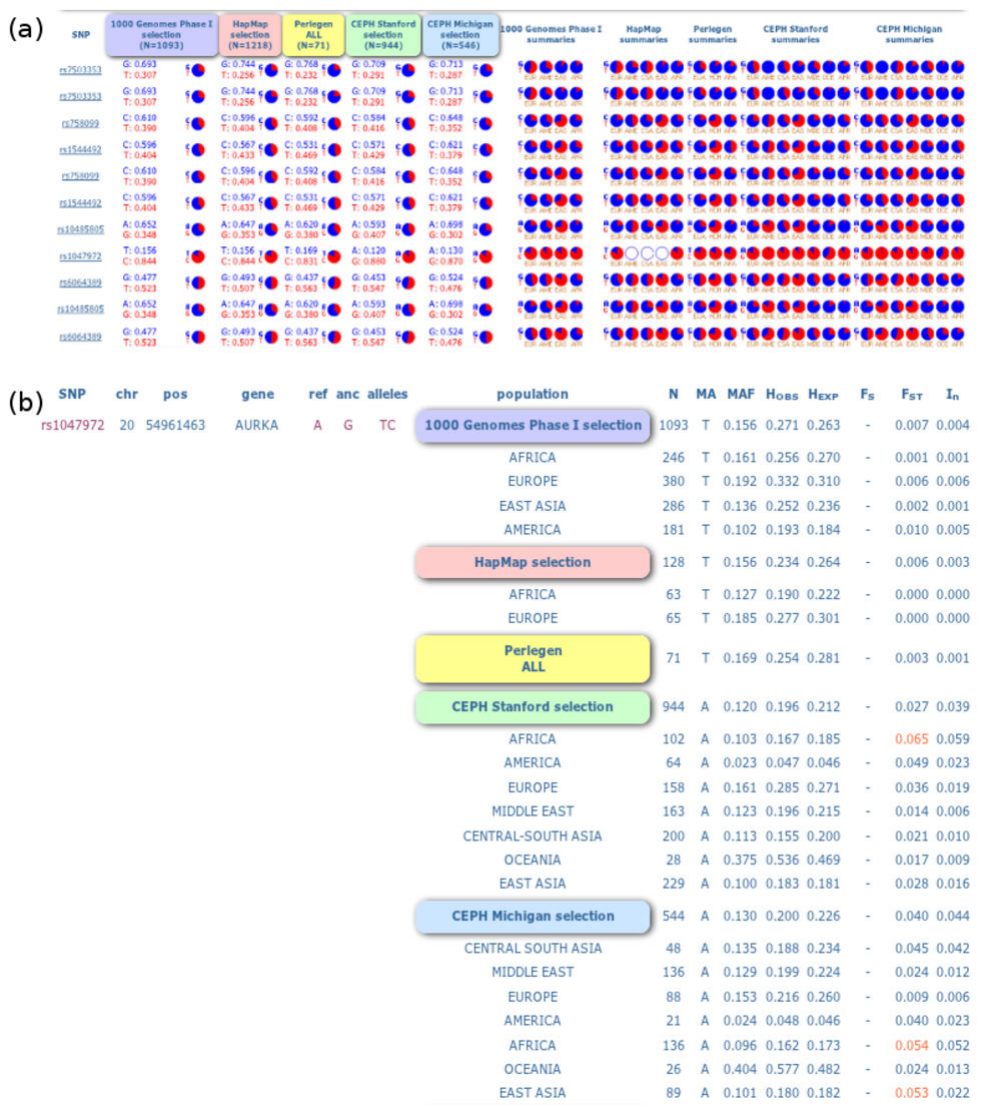
Results from population genetics analysis of Aurora kinases. (a) Frequency distribution of Aurora-A single neucleotide polymorphism’s (SNPs) in different human population. (b) Heterozygosity values of the minor and major alleles in different human population

**Figure 5:**
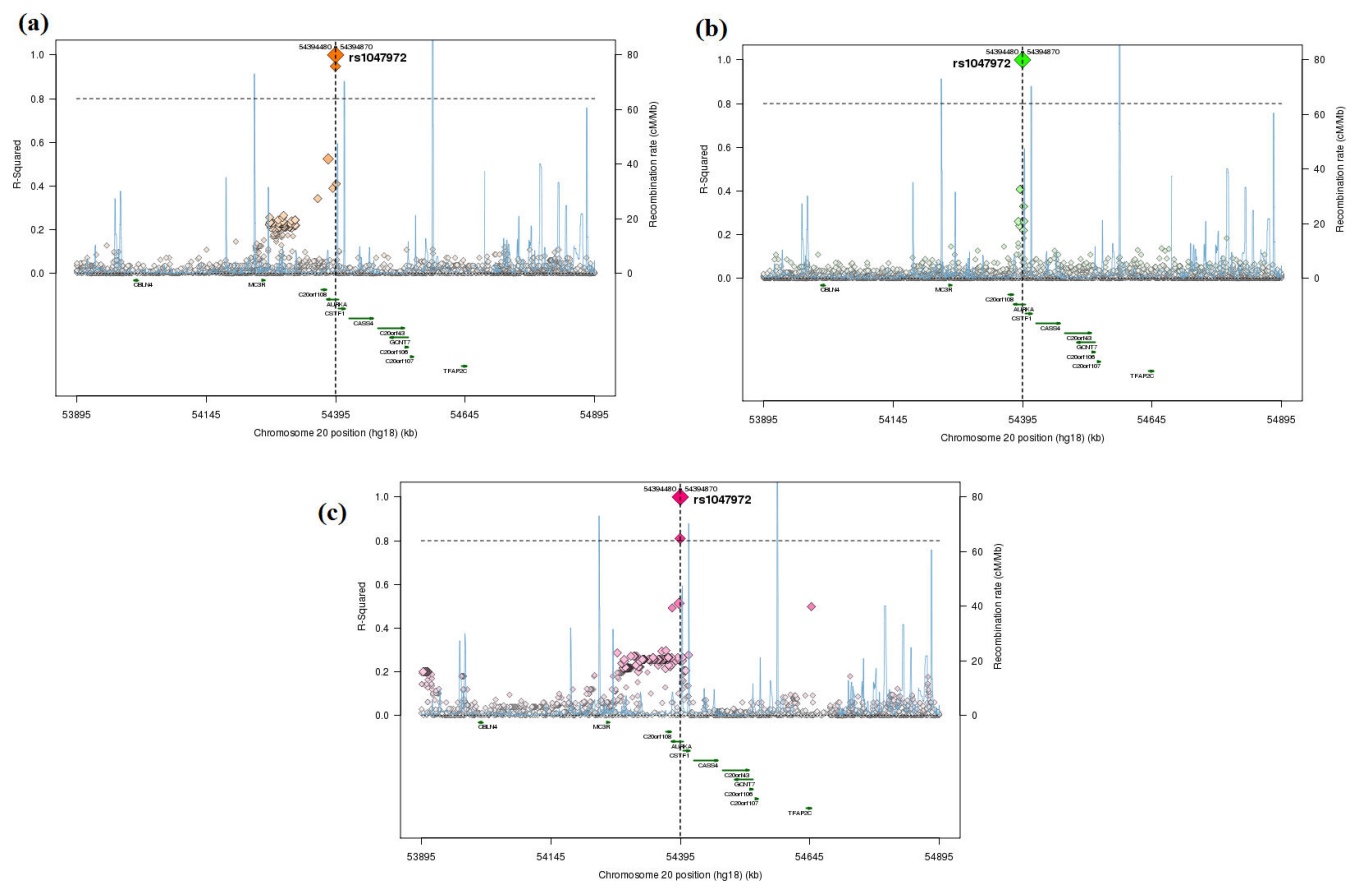
The R-Squared values and recombination rates of rs1047972 in (a) CEU; (b) YRI and (c) CHB/JPT populations.

We further extended our observation to the evolutionary selection of Aurora kinases occurring in the current human population. In the process of substitution, a previously non-existent allele arises by mutation and undergoes fixation by spreading through the population by random genetic drift and/or positive selection. Once the frequency of the allele is at 100%, it is said to be fixed in the population. Detecting the possibility of mutation fixation rates might provide a clear picture of current evolutionary trend followed by aurora kinases in human. I57V mutation in Aurora-A was shown to be highly penetrant, prevailing in 84.4% of human populations (Figure 4a-b). This directly showed that the mutant allele had been strongly under the influence of evolutionary selection and had invaded up to a very large mass of human population. To examine the molecular effect of the mutation, we further subjected it to SNPeffect4.0, I-mutant2.0, SIFT, Polyphen and MutPred tools. No damaging or deleterious consequences were observed in SIFT and polyphen scores. Moreover, SNPeffect4.0 depicted no change in LIMBO, TANGO and WALTZ score, suggesting no impact on the aggregation tendency, chaperone binding tendency and the amyloid propensity of protein respectively. The results clearly suggested that the mutation had been completely accepted in a large mass of human population genome, in terms of its functional and structural effects and did not cause any negative consequences on the protein activity. Furthermore, the I-mutant2.0 scores suggested that the free energy change induced by the mutations were also tolerable, indicating negligible tendency of mutation to cause structural damages. Further, we implemented MutPred tool to examine the molecular changes invoked by the I57V mutation. It was interesting to observe that the mutation has tendency to invoke three significant changes in the protein, including gain of Molecular Recognition Features (MoRFs) binding, gain of phosphorylation at S53 amino acid position and gain of glycosylation at P58. MoRFs were short, interaction-prone segments of protein disorder that undergo disorder-to-order transitions upon specific binding, representing a specific class of intrinsically disordered regions that exhibited molecular recognition and binding functions. MoRFs were common in various proteomes and occupy a unique structural and functional niche in which function was a direct consequence of intrinsic disorder. Gain of MoRF binding would significantly aid in scaffold regulation of aurora kinase A. Furthermore, gain of phosphorylation and glycosylation at the corresponding residues also presented an insight of the gain of functional properties induced by the mutation. Moreover, this mutation seemed to cross the fixation threshold and could be an essential part of the Aurora-A evolutionary road map. The observations indicated strong fixation tendency of mutant allele and was further confirmed by the linkage disequilibrium scores obtained from the SNAP tool (Figure 5). Figure 5 showed that the mutation had very high linkage disequilibrium scores and recombination rates in Utah residents with ancestry from northern and western Europe (CEU), Yoruba (YRI), Han Chinese in Beijing (CHB) and Japanese in Tokyo (JPT) populations. Higher recombination rates of alleles were generally useful to introduce genetic novelty into the existing genotypes, which further explained the chances for the selection of I57V mutation in Aurora-A kinase. The overall results strongly suggested that the Aurora A I57V mutation had deeply invaded the human population and was likely to induce significant gain in the functional activity of protein. It would be interesting to examine the molecular and phenotypic changes associated with I57V mutation and its benefits.
